# Community-based Randomized Controlled Trial of Non-pharmacological Interventions in Prevention and Control of Hypertension among Young Adults

**DOI:** 10.4103/0970-0218.58393

**Published:** 2009-10

**Authors:** LG Saptharishi, MB Soudarssanane, D Thiruselvakumar, D Navasakthi, S Mathanraj, M Karthigeyan, A Sahai

**Affiliations:** Department of Preventive and Social Medicine, Jawaharlal Institute of Post-graduate Medical Education & Research (JIPMER), Puducherry-6, India

**Keywords:** Community-based randomized controlled trial, hypertension, relative efficacies, physical exercise, salt intake reduction, yoga, young adults

## Abstract

**Context:**

Hypertension is a major chronic lifestyle disease. Several non-pharmacological interventions are effective in bringing down the blood pressure (BP). This study focuses on the effectiveness of such interventions among young adults.

**Aims:**

To measure the efficacy of physical exercise, reduction in salt intake, and yoga, in lowering BP among young (20-25) pre-hypertensives and hypertensives, and to compare their relative efficacies.

**Settings and Design:**

The study was done in the urban service area of JIPMER. Pre-hypertensives and hypertensives, identified from previous studies, constituted the universe. The participants were randomized into one control and three interventional groups.

**Materials and Methods:**

A total of 113 subjects: 30, 28, 28 and 27 in four groups respectively participated for eight weeks: control (I), physical exercise (II) - brisk walking for 50-60 minutes, four days/week, salt intake reduction (III) - to at least half of their previous intake, and practice of yoga (IV) - for 30-45 minutes/day on at least five days/week.

**Statistical Analysis Used:**

Efficacy was assessed using paired t test and ANOVA with Games Howell post hoc test. An intention to treat analysis was also performed.

**Results:**

A total of 102 participants (29, 27, 25 and 21 in groups I, II, III and IV) completed the study. All three intervention groups showed a significant reduction in BP (SBP/DBP: 5.3/6.0 in group II, 2.6/3.7 in III, and 2.0/2.6 mm Hg in IV respectively). There was no significant change (SBP/DBP: 0.2/0.5 mmHg) of BP in control group (I). Physical exercise was most effective (considered individually); salt intake reduction and yoga were also effective.

**Conclusions:**

Physical exercise, salt intake reduction, and yoga are effective non-pharmacological interventions in significantly reducing BP among young hypertensives and pre-hypertensives. These can therefore be positively recommended for hypertensives. There is also a case to deploy these interventions in the general population.

## Introduction

Hypertension is a major chronic lifestyle disease and an important public health problem worldwide. A recent report indicates that nearly one billion adults had hypertension in 2000, and this is predicted to increase to 1.56 billion by 2025.(1) This leads to numerous micro/macro vascular complications and subjects with hypertension are known to have a two-fold higher risk of developing coronary artery disease, four times higher risk of congestive heart failure and seven times higher risk of cerebrovascular disease compared to normotensive subjects.(2) Hence, it is important to maintain BP within the normal range.(3)

There are several non-pharmacological methods of controlling blood pressure (BP).(4) physical activity,(5–7) yoga,(8) relaxation techniques(9–11) and reduction in daily salt intake, have been proven to be of use. These interventions modify the risk factors responsible for the development of hypertension. Earlier studies in Puducherry have also highlighted the role of the above risk factors in the development of elevated blood pressure.(12,13)

This study focuses on tackling these important risk factors of hypertension among *young adults* through non-pharmacological interventions at an early stage of the natural history of the disease, and compares their relative efficacies in reducing BP and hence the objectives,

1. To measure the efficacy of the following interventions in lowering BP among young (20-25) pre- hypertensives and hypertensives:
  - Physical exercise
  - Reduction in salt intake and
  - Practice of yoga
2. To compare the relative efficacies of these three interventions.

## Materials and Methods

The study was done in Kuruchikuppam, an urban service area of Department of P and SM, JIPMER. The universe of the study was the 224 confirmed hypertensives/pre-hypertensives from the cohort studied earlier between 2002-06.(12,13) This included **51** hypertensives and **173** pre-hypertensives, as per the JNC VII criteria for classification of pre-hypertension (120-139 mm Hg SBP and/or 80-89 mm Hg DBP).(14) Since it was not feasible to cover this high number within the study period of two months an operational definition for pre-hypertension as SBP 130-139 mm Hg and/or DBP 85-89 mm Hg was used,. Thus the sample worked out to 136 (51 hypertensives plus 85 pre-hypertensives). Twelve members (five hypertensives plus seven pre-hypertensives) of the proposed sample could not be traced as they had shifted out of the service area. Of the 46 hypertensives, fourwere excluded (as advised by the Institute Ethics Committee) as they had severe hypertension. The remaining 120 subjects were divided into one control and three interventional groups of 30 each, using a standardized randomization process, with a random number generator (SPSS 13.0).

Of the 120, seven participants (three hypertensives plus four pre-hypertensives) did not consent to be part of the program, while 30, 28, 28 and 27 in the control, physical exercise, salt intake reduction and yoga groups respectively consented amounting to a total of 113 study subjects [39 hypertensives: nine, 12, eight and 10 in the Control (Group I), physical exercise (Group II), salt intake reduction (Group III) and yoga (Group IV) respectively]. The pathogenesis, risk factors, complications and therapy of hypertension was explained to the participants in the local language, Tamil, so as to motivate them for compliance. Their blood pressure was measured at the start of the study period using the mercury sphygmomanometer.

### Physical exercise group (Group II)

Participants of this group were motivated to undergo physical exercise in the form of brisk walking for 50-60 minutes, four days per week.(15–17) As a way of encouraging them, the investigator accompanied them on several occasions.

### Salt intake reduction group (Group III)

These subjects were motivated to reduce their daily salt intake to at least half of their previous intake.(15,18,19) Data on their previous intake was available from the earlier studies.(12,13) The participants were given practical suggestions on reducing their salt intake. They were also asked to use separate salt packets and their food was cooked separate from that of other family members. The ‘questionnaire method’ was used for assessing compliance. The participants were requested to record details of their salt intake and the extent of their adherence to the instructions given. Fortnightly random quantification of salt intake was done using the feedback from participants.

### Yoga group (Group IV)

This group of participants was taught yoga (*asanas* proved to be effective in BP reduction(20,21)) by a qualified yoga teacher of the institute. They were encouraged to practice yoga for 30-45 minutes per day for at least five days a week.(15) This included relaxation techniques like *pranayama* (breathing exercises); and *asanas* like *savasana, ardha matsyendrasana, naadishudhi asan*a, single leg, and double leg raise.

All these interventions were carried out for eight weeks. Flexibility was allowed for those who took time for motivation and started the intervention at subsequent dates; however they were followed up for the corresponding eight-week period. At the end of the study period their BP values were measured.

Out of the initial 113, 11 subjects (including 6 hypertensives) dropped out. At the end of the study 29, 27, 25 and 21 in the groups I, II, III and IV respectively (102 comprising 33 hypertensives: eight, 12, six and seven in the groups I, II, III and IV respectively) were successfully followed through.

The study was approved by the Institute Research Council and Institute Ethics Committee. Informed consent was obtained from all the participants.

### Statistical analysis

The pre-intervention and the post-intervention BP values were analyzed using the paired ‘t’ test. ‘Intention to treat’ analysis was carried out by including the attrition cohort. The inter-group comparisons were made using ANOVA with Games Howell post hoc test.

## Results

Of the 102 participants who completed the study, 68 were males and 34 females. Age distribution concurred in all groups. The male-female distribution was similar in all groups except a slight over-representation in the salt intake reduction group. During course of the study one, one, three and five participants dropped out from groups I, II, III and IV respectively. The mean systolic BP in the four groups ranged between 123 and 128 mm Hg and the diastolic blood pressure between 82 and 87 mm Hg. Considering those who completed the study, the baseline mean SBP and DBP of the four groups differed significantly; this could be due to their differential attrition patterns. The distribution of hypertensives among the groups is also significantly different due to the effect of attrition [Table 1].

**Table 1:** Baseline characteristics of the study population

Baseline Characteristics	Total study group	Control group	Physical exercise group	Salt intake reduction group	Yoga group
Number at the start (n)	113	30	28	28	27
Number at the end (n)	102	29	27	25	21
Attrition rate (%)	9.7	3.3	3.5	10.7	22.2
Age (mean±SD)*	22.5 ± 1.3	22.5 ± 1.4	22.4 ± 1.3	22.5 ± 1.47	22.5 ± 1.36
Male/ female ratio*	68/34	20/9	19/8	15/10	14/7
Number of hypertensives* (n)	33	8	12	6	7
Systolic blood pressure* (mean±SD)	125.5 ± 9.3	123.1 ± 10.2	128.6 ± 7.7	124 ± 8.1	126.8 ± 10.3
Diastolic blood pressure* (mean±SD)	84.6 ± 6.5	82.9 ± 7.1	87.4 ± 4.8	83.7 ± 6.7	84.5 ± 6.5

* per protocol group

As regards SBP in the pre and post intervention setting, in the physical exercise group there was a reduction from 128.6 to 123.3 (5.3), in the salt intake reduction group from 124.0 to 121.4 (2.6), in the yoga group from 126.8 to 124.8 (2.0), and in the control group from 123.1 to 122.9 (0.2). This fall in SBP in each of the interventional groups was statistically significant (*P* less than 0.05 in each case) compared to the control group wherein the fall was not significant [Table 2]. Likewise for DBP, the physical exercise group showed a reduction from 87.4 to 81.4 (6.0), salt intake reduction group from 83.7 to 80.0 (3.7), yoga group from 84.5 to 81.9 (2.6), and control group from 82.9 mm Hg to 82.4 mm Hg (0.5). This reduction in the DBP values in all the three interventional groups was statistically significant (*P* less than 0.05 in each case) whereas the change in the DBP values of the control group was not significant [Table 3]. ‘Intention to treat analyses’ also revealed statistically significant reduction in systolic and diastolic blood pressure values in all the three interventional groups.

**Table 2:** Comparison of pre and post intervention systolic blood pressure (SBP) values

Groups	Systolic blood pressure analysis					
	
	Per protocol analysis			Intention to treat analysis		
		
	Pre (mean±SD)	Post (mean±SD)	95% confidence interval	Pre (mean±SD)	Post (mean±SD)	95% confidence interval
Control	123.1 ± 10.2	122.9 ± 9.7	-0.52 to 0.73	123.8 ± 10.8	123.7 ± 10.4	-0.5 to 0.70
Physical exercise	128.6 ± 07.7	123.3 ± 5.3	3.62 to 6.90	128.4 ± 7.6	123.3 ± 5.2	3.44 to 6.70
Salt reduction	124.0 ± 08.1	121.4 ± 6.8	1.33 to 3.95	123.1 ± 8.1	120.8 ± 6.7	1.15 to 3.56
Yoga	126.8 ± 10.3	124.8 ± 9.3	0.64 to 3.36	127.6 ± 9.9	126.0 ± 9.3	0.47 to 2.64

*paired ‘t’ test

**Table 3:** Comparison of pre and post intervention diastolic blood pressure (DBP) values

Groups	Diastolic blood pressure analysis					
	
	Per protocol analysis			Intention to treat analysis		
		
	Pre (mean±SD)	Post (mean±SD)	95% confidence interval	Pre (mean±SD)	Post (mean±SD)	95% confidence interval
Control	82.9 ± 7.1	82.4 ± 7.2	-0.26 to 1.1	83.2 ± 7.2	82.8 ± 6.3	-0.26 to 1.1
Physical exercise	87.4 ± 4.8	81.4 ± 4.0	4.89 to 7.19	87.4 ± 4.8	81.6 ± 4.0	4.63 to 7.01
Salt reduction	83.7 ± 6.7	80.0 ± 4.8	2.64 to 4.72	83.7 ± 6.8	80.3 ± 5.3	2.26 to 4.31
Yoga	84.5 ± 6.5	81.9 ± 5.3	1.67 to 3.57	85.8 ± 6.7	83.8 ± 6.3	1.19 to 2.88

*paired ‘t’ test

After checking for normality using Kolmogrov-Smirnov test, ANOVA was done with Games-Howell post hoc test as Levene's test for equal variance was significant. On analysis of the relative efficacies, considering SBP, there were statistically significant differences with Physical exercise Vs salt intake reduction (*P* is equal to 0.01) and physical exercise Vs practice of yoga (*P* is equal to 0.009). However, when the effectiveness of salt intake reduction and yoga were compared, there was no significant difference [Table 4]. Similarly for DBP, Physical exercise vs salt intake reduction (*P* is equal to 0.002) and Physical exercise vs practice of yoga (*P* is equal to 0.000) showed statistically significant differences. Here too, salt intake reduction Vs practice of yoga showed no significant difference [Table 5].

**Table 4:** Relative efficacies of the three interventions in reduction of systolic blood pressure (SBP) - physical exercise, salt intake reduction and yoga

Group A	Group B	Mean difference (A-B)	Sig.	95% confidence interval	

				Lower bound	Upper bound
Walking	Yoga	3.479*	0.002	1.16	5.79
	Salt	2.942*	0.010	0.61	5.28
Yoga	Walking	-3.479*	0.002	-5.79	-1.16
	Salt	-0.536	0.766	-2.39	1.31
Salt	Walking	-2.942*	0.010	-5.28	-0.61
	Yoga	0.536	0.766	-1.31	2.39

* ANOVA with Games-Howell post hoc test

**Table 5:** Relative efficacies of the three interventions in reduction of diastolic blood pressure (DBP) - physical exercise, salt intake reduction and yoga

Group A	Group B	Mean difference (A-B)	Sig.	95% confidence interval	
				
				Lower bound	Upper bound
Walking	Yoga	3.747*	0.000	2.01	5.48
	Salt	2.886*	0.001	1.05	4.73
Yoga	Walking	-3.747*	0.000	-5.48	-2.01
	Salt	-0.861	0.391	-2.43	0.71
Salt	Walking	-2.886*	0.001	-4.73	-1.05
	Yoga	0.861	0.391	-0.71	2.43

* ANOVA with Games-Howell post hoc test

An attrition rate of 9.7% (3.3, 3.5, 10.7 and 22.5% in the groups I, II, III and IV respectively) was recorded. This study found physical exercise and salt restriction to be more acceptable than yoga at the community level.

## Discussion

This study has highlighted the effectiveness of physical exercise, salt intake reduction and yoga in prevention and control of hypertension among young adults. A comparative discussion of these variables follows.

### Physical exercise

There are several studies comparable with the present study. Hagberg *et al*.(17) report that exercise training decreases blood pressure (BP) in approximately 75% of individuals with hypertension, with systolic and diastolic BP reductions averaging approximately 11 and 8 mm Hg, respectively. Chiriac *et al*.(6) found that in addition to preventing hypertension, regular exercise lowers BP (10 mm Hg SBP/DBP), improves lipoprotein lipid profiles and insulin sensitivity. de Luis *et al*.(18) record a 4.6 mm Hg reduction in BP with aerobic exercise. Schwarz *et al*.(5) have shown that regular sports activity of moderate intensity suffices to bring about a lowering of blood pressure. Anand MP,(15) through his meta-analysis of 23 RCTs, showed that moderately intense exercise, e.g., 30 to 45 minutes of brisk walking on four days a week, lowers BP.

Our study broadly concurs with the ‘Lifestyle modifications to prevent and control hypertension’ study, wherein, Cleroux *et al*.(16) presented the following recommendations with regard to physical exercise: (1) People with mild hypertension should engage in 50-60 minutes of moderate rhythmic exercise of the lower limbs, such as brisk walking or cycling, three/four times per week to reduce blood pressure (2) Exercise should be prescribed as an adjunctive therapy for people who require pharmacologic therapy for hypertension (3) People who do not have hypertension should participate in regular exercise as it will decrease blood pressure and reduce the risk of coronary artery disease.

Whelton *et al*.,(22) through their meta-analysis of 54 RCTs, conclude that aerobic exercise reduces blood pressure in both hypertensive and normotensive persons. They have shown a net reduction of 3.8 and 2.6 mm Hg in systolic and diastolic blood pressures respectively and, further go on to add that an increase in aerobic physical activity should be considered an important component of lifestyle modification for prevention and treatment of high blood pressure. All the aforementioned studies concur with our study with regards to the efficacy of physical exercise.

It is to be noted that the above studies, including the present one, deal with moderately intense exercise as an intervention. On the other hand, Cooper *et al*.(7) concluded that despite high compliance with moderate intensity exercise program, the magnitude of its BP lowering effect was not as great as that found in studies of higher intensity exercise (aerobic) among hypertensives. This study differs with rest of the literature available in this regard.

### Salt intake reduction

A reduction in the dietary salt intake as a strategy to tackle hypertension has been tried in various studies. Anand,(15) through his meta-analysis of 23 RCTs showed that a 100 mmol/day reduction in sodium intake was associated with a decline of five to seven mm Hg (systolic)/2.7 mm Hg (diastolic) in hypertensive subjects. de Luis *et al*.(18) recorded a 3.6 mm Hg reduction in BP following reduction in dietary salt.

In a Cochrane systematic review by Hooper *et al*.(23) (including 17 trials in individuals with elevated blood pressure and 11 trials in individuals with normal BP) a modest reduction in salt intake for a duration of 4 weeks or more was found to have a significant and, from a population viewpoint, important effect on blood pressure. This meta-analysis also demonstrated a correlation between the magnitude of salt reduction and the magnitude of BP reduction, within the daily intake range of three to 12 g/day of salt. This finding correlates with that of the present study.

Another meta-analysis by He *et al*.(24) reviewed the results of all unconfounded randomized trials aiming to reduce sodium intake in healthy adults over at least six months. Systolic and diastolic BP were reduced (systolic by 1.1 mmHg and diastolic by 0.6 mmHg) at 13-60 months. This study did not find any correlation between the degree of reduction in sodium intake and change in BP.

Cappuccio(25) comments that reducing salt intake improves cardiovascular outcomes and goes on to recommend that countries should come up with effective policies to regulate salt intake in the community. Lawrence J Appel(26) recommends halving of salt usage in processed and restaurant food. Our findings add strength to the view put forth by the above mentioned commentaries.

### Practice of yoga

Yoga has been proven to be highly effective in reducing BP by numerous Indian as well as international studies. Singh *et al*.(27) reported a significant reduction in BP (12 mm Hg in SBP; 11.2 mm Hg in DBP) with a 40-day yoga regimen among Type 2 diabetics.

Schwickert *et al*.(9) and Frumkin *et al*.(4) consider yoga to be a relaxation technique that is highly effective in reduction of elevated BP and management of stress. Bijlani *et al*.(8) concluded that a short lifestyle modification and stress management education program (with yoga as the major component) led to favorable metabolic effects within nine days. Aivazyan *et al*.(10) demonstrated a significant reduction in SBP and DBP, peripheral vascular resistance, and hypertensive response to emotional stress, and an improvement in psychological adaptation, quality of life, and capacity for work. This study concurs with these findings supporting yoga as an effective intervention.

### Comparison of their relative efficacies

This study showed that physical exercise was more effective than the other two interventions (considered individually) whereas both salt intake reduction and yoga were equally effective as non-pharmacological interventions for prevention and control of hypertension among young adults. In a recent meta-analysis, de Luis *et al*.(17) demonstrated reduction in BP of 4.6 mmHg with aerobic exercise and 3.6 mmHg with decrease in salt intake; this finding is comparable with findings of this study.

### Acceptability of the interventions

Comparable attrition rates (3%) in the physical exercise and the control groups highlight the high acceptability of physical exercise. Salt restriction group also registered a high acceptability of 90%. Even in the yoga group, the acceptability was as high as 78%.

The WHO document on ‘Community Prevention and Control of Cardiovascular Diseases’(28) provides estimates that a 2 mm Hg downward shift in the entire distribution of SBP in the community is likely to reduce the annual mortality from stroke by six per cent, coronary heart disease by four per cent, and all causes by three per cent. The corresponding benefits for a 3 mm Hg downward shift in SBP have been estimated to be eight, five and four per cent respectively. This stresses the importance of such non-pharmacological interventions at the community level. This study therefore, adds to the existing literature on the utility of major non-pharmacological interventions (daily walking, reduction in salt intake and yoga) in reduction of elevated blood pressure and, hence the morbidity and mortality associated with stroke and coronary heart disease in the community.

### Limitations

An attrition rate of 9.7% (3.3, 3.5, 10.7 and 22.5% in the groups I, II, III and IV respectively) was recorded during the course of this study.

The study assumed that all participants underwent the same magnitude of intervention. There could have been variations in the extent of compliance among members of the same interventional group. The study did not have any mechanism to standardize or quantify the actual magnitude of intervention per individual.
